# Relationship and Training Effects of Horizontal Multi-Step Jumps on Sprint Performance: A Systematic Review

**DOI:** 10.3390/jfmk11010095

**Published:** 2026-02-26

**Authors:** Bjørn Johansen, Roland van den Tillaar

**Affiliations:** Department of Sports Sciences and Physical Education, Nord University, 7600 Levanger, Norway; 06091696@student.nord.no

**Keywords:** correlation, training transfer, plyometric training, bounding, single-leg jumps, ground contact time, kinematic variables

## Abstract

**Background:** This systematic review examined the relationship between horizontal multi-step jumps and sprint performance, and whether training interventions including these exercises improve sprinting. **Methods:** A systematic literature search was conducted in SPORTDiscus and PubMed (MEDLINE) and included English-language studies of athletes aged ≥14–15 years that assessed at least one horizontal multi-step jump and reported sprint outcomes over distances up to 100 m. Methodological quality and risk of bias were assessed using design-appropriate critical appraisal tools. Of 316 records identified, 19 studies met the inclusion criteria (10 intervention studies and 9 correlational studies). **Results:** Across correlational studies, horizontal multi-step jump performance showed associations ranging from weak to very large with sprint performance, with the strongest relationships typically observed during acceleration (≤20–30 m). In trained sprinters, correlations were often large to very large (r ≈ −0.65 to −0.88), whereas team-sport athletes showed more moderate associations, and younger or less specialized populations showed weak or non-significant relationships. Across intervention studies, horizontal multi-step jump training generally improved short-distance sprint performance, with the largest improvements reported for acceleration (up to ~7–12% in some studies), while effects at longer sprint distances and maximal-speed performance were smaller, inconsistent, or not different from comparison training. **Conclusions:** Overall, the evidence suggests that the association between horizontal multi-step jumps and sprint performance is strongest during the acceleration phase and is influenced by athlete population and training status. Horizontal multi-step jumps appear to be useful for assessing and potentially developing sprint acceleration. However, the findings should be interpreted with caution due to heterogeneity in study design and variable methodological quality, and associations with maximal sprint speed are less consistent across studies.

## 1. Introduction

Sprint speed in sprint in both athletics and team sports is largely determined by the athlete’s ability to develop high horizontal force over short contact times, especially during the acceleration phase [1,2]. Contact time and the direction and magnitude of ground reaction forces are key kinematic and kinetic determinants of sprint performance across both acceleration and maximal-speed phases, as even small changes in these variables can meaningfully influence running velocity [3,4]. As a result, tests and training methods that reflect the horizontal and rapid force demands of sprinting have received increasing research attention [5]. Horizontal multi-step plyometric exercises, such as bounding and various forms of single-leg tasks, are often highlighted as sprint-relevant because they require forward-directed force production under temporal constraints that are comparable to sprinting [5,6]. The direction of force application is also known to influence the transfer of training to sprint performance, particularly in early acceleration [5,7]. For these reasons, horizontal jump tasks are now widely used both as performance assessments and as training exercises in sports where rapid horizontal displacement is essential [8,9].

The performance and mechanical demands of horizontal multi-step jumps vary, as factors such as step pattern: single- or double-leg execution, and whether the goal is speed or distance may influence which kinematic strategies the athlete uses. Previous studies have shown that such differences can result in varying associations with sprint performance, with some horizontal jump types showing strong relationships with sprint speed, while others show weaker or more inconsistent findings [6,9]. This suggests that different types of horizontal jumps do not fully capture the same sprint-relevant properties, and it remains unclear which specific multi-step jumps best reflect the kinematic and mechanical determinants of sprinting. Recent research also suggests that different horizontal jumps may highlight different sprint-relevant capacities, with some exercises appearing to be more closely related to sprint kinematic variables than others [10,11]. At the same time, intervention studies show that the response to such jump-based training can vary significantly between exercises and athletes [6,11]. Thereby, there is no comprehensive overview summarizing which types of horizontal multi-step jumps have the strongest relationship to sprint performance, or which horizontal jump-based training interventions actually lead to improvements in 0–100 m sprint.

Therefore, the purpose of this systematic review is to compile and synthesize existing research investigating the relationship between horizontal multi-step jumps and sprint performance over 0–100 m, and to evaluate the reported effects of jump-based training interventions in which such jumps constitute a significant component of the training. The review includes both correlational and intervention studies and focuses on rectilinear horizontal multi-step jump tasks (e.g., bounding and single-leg jumps) that are biomechanically relevant to sprinting.

Specifically, this review addresses the following questions:

(1) What is the reported relationship between horizontal multi-step jumps and sprint performance across different athlete populations and sprint phases?
(2) Do training interventions including horizontal multi-step jumps improve sprint performance, and if so, over which sprint distances or phases are effects most consistently observed?

## 2. Materials and Methods

### 2.1. Search Strategy

This systematic review aimed to summarize studies examining the relationship between horizontal multi-step jumps and sprint performance, as well as training interventions in which such jumps form a central component. A systematic literature search was conducted in SPORTDiscus and PubMed (MEDLINE) from database inception at 1 October 2025. These databases were selected because they are widely used in sports science and exercise physiology and cover most of the peer-reviewed literature in this field. Scopus and Web of Science were not included due to substantial overlap in indexed journals and feasibility considerations.

Search terms were combined using Boolean operators (AND/OR). All identified records were imported into Rayyan for screening. To ensure that no relevant studies assessing sprint performance over longer distances were overlooked, targeted supplementary searches were subsequently conducted in PubMed and SPORTDiscus using modified search terms focusing on sprint distances beyond 40 m. These supplementary searches did not identify any additional eligible studies. The exact search strings used in each database were as follows:

In PubMed (MEDLINE), the following terms were combined: standing broad jump OR standing long jump OR single-leg hop OR triple hop OR crossover hop OR bounding OR multiple hop* OR five bound OR 5-bound OR hop test*, AND sprint OR 20 m OR 30 m OR 40 m OR 0–40 m OR linear speed OR velocity, AND correlation OR relationship OR association OR randomized OR intervention OR training. The search was limited to English language publications and participants aged 14 years and older.

In SPORTDiscus with Full Text, the following search string was used: TX (standing broad jump OR standing long jump OR “single-leg hop” OR “triple hop” OR “crossover hop” OR bounding OR “multiple hop*” OR “5-bound” OR “five bound” OR “hop test*”) AND TX (sprint OR “10 m” OR “20 m” OR “30 m” OR “40 m” OR “linear speed”) AND TX (correlation OR relationship OR intervention OR randomized OR training) AND LA (English) AND (AG 14–17 OR AG 18–64).

### 2.2. Eligibility Criteria

Studies were included if they met the following criteria: (i) they were published in English and peer-reviewed; (ii) they included athletes aged 14–15 years or older; (iii) they employed at least one horizontal multi-step jump task, such as bounding, single-leg jumps for speed or distance, standing triple jump, five-jump tests, or repeated hops; and (iv) they reported sprint performance outcomes over distances of up to 100 m, including split times where applicable, either as performance relationships with horizontal jump measures or as training-induced changes following an intervention.

Lateral or non-linear jumps were excluded. Eligible study designs included either correlational studies reporting associations between horizontal jump performance and sprint outcomes, or intervention studies in which horizontal multi-step jumps constituted a main component of the training program. Reviews, modelling studies without performance data, non-English publications, and studies lacking relevant sprint outcomes were also excluded. This systematic review was conducted and reported in accordance with the PRISMA 2020 guidelines [12].

### 2.3. Study Selection

**Figure 1:**
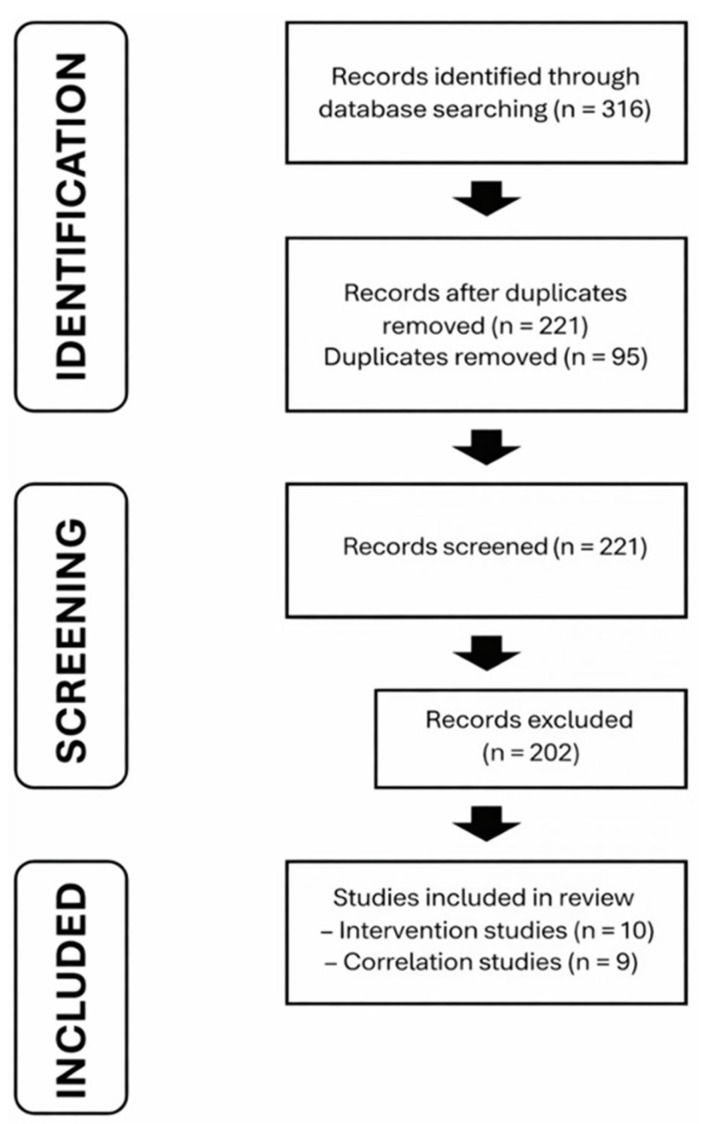
PRISMA flow diagram of the study selection process.

The initial database search yielded 316 records. After removal of duplicates, 221 articles remained for title and abstract screening, of which 202 were excluded. The full texts of the remaining studies were assessed for eligibility, resulting in a final inclusion of 19 studies: 9 correlational and 10 intervention studies (Figure 1).

### 2.4. Data Extraction

Data was extracted from each included study using a structured Excel spreadsheet. Data extraction was performed by one reviewer and subsequently checked for consistency and completeness by a second researcher. Extracted variables included authorship, publication year and journal, study design (intervention or correlational), participant characteristics (age, sex, sport, and sample size), type and description of the horizontal jump tasks, sprint test distances and timing methods, intervention duration and weekly training frequency, and sprint performance outcomes over distances of up to 100 m. For correlational studies, reported associations between horizontal jump performance and sprint outcomes were extracted, whereas for intervention studies, pre–post changes and between-group comparisons were recorded. Between-group outcomes were summarised using short, standardised descriptive statements. The magnitude of correlation coefficients was interpreted using conventional thresholds, with coefficients of ~0.10–0.29 considered weak, ~0.30–0.49 moderate, and ≥0.50 strong, in line with commonly used guidelines in sports science [13]. A meta-analysis was not performed due to significant heterogeneity in study design, participant characteristics, horizontal jumping tasks, sprint outcome measures, and intervention protocols. This systematic review was not prospectively registered.

### 2.5. Methodological Quality Assessment

The methodological quality of the included studies was assessed using structured checklists developed for the purpose of this review, based on commonly accepted methodological criteria for cross-sectional and intervention study designs.

The checklists were constructed based on established methodological criteria commonly applied in sports science research, including participant selection, measurement validity, statistical reporting, and transparency of intervention procedures. The assessment was performed by one reviewer and discussed with the second author in cases of uncertainty.

For correlational studies, an eight-item checklist was used to evaluate participant selection, clarity of inclusion criteria, validity and reliability of measurement procedures, identification and consideration of potential confounding variables, and appropriateness of statistical analyses.

For intervention studies, a nine-item checklist was applied, including the above criteria and additional aspects such as allocation procedures, presence of a comparison or control group, and completeness of outcome reporting.

Each item was rated as “yes” or “no”. Based on the total number of positive ratings (yes total), studies were classified as low risk of bias (7–8/8 for correlational studies; 8–9/9 for intervention studies), moderate risk of bias (4–6 positive ratings), or high risk of bias (≤3 positive ratings).

## 3. Results

Nineteen studies met the inclusion criteria, including ten intervention studies and nine correlational studies (Figure 1). Participants ranged from early-adolescent to adult athletes across football, handball, rugby, sprinting, and mixed-sport groups. A variety of horizontal multi-step jump tests were used (e.g., bounding, standing triple jump, five-jump tests, and single-leg horizontal hops), and sprint performance was assessed over distances ranging from 5 to 100 m, with most studies focusing on sprint acceleration within the first 20–30 m. Most studies used electronic timing systems, although manual timing methods were also employed in a small number of studies. The methodological quality assessment indicated that most correlational studies were classified as moderate risk of bias, whereas intervention studies were predominantly classified as low to moderate risk (Table 1).

Across the nine correlational studies, associations between horizontal multi-step jump performance and sprint performance were examined, with mixed findings across studies (Table 2). The strongest relationships were observed in trained sprinters, where horizontal multi-step jump tests showed large to very large correlations with sprint acceleration (e.g., r = −0.65 to −0.88 for 10–30 m sprint performance) [16]. Similar findings were reported by [22], who observed very large correlations between horizontal jump performance and 100 m sprint time (r = −0.88). Moderate to strong associations were also reported in team-sport athletes; for example, standing triple jump and single-leg horizontal jump tests were moderately to strongly correlated with 10–20 m sprint performance, r = 0.50–0.75 [15,17,18]. In contrast, weaker or non-significant associations were reported in younger or less specialized populations, including youth soccer players, where horizontal jump measures showed only trivial to small correlations (r = 0.18–0.24) with sprint performance [19].

Moderate correlations (approximately r = 0.40–0.55) were observed in several mixed-sport and youth cohorts [14,18,21] (Table 2), whereas one study examining horizontal jump asymmetries reported weak and non-significant associations with sprint performance [19]. Across correlational studies, horizontal multi-step jump tasks involving alternating ground contacts tended to show stronger and more consistent associations with sprint performance than isolated single-leg horizontal jump tests, although both jump types were primarily related to sprint acceleration outcomes (≤20 m) (Table 2). Several of the included correlational studies were based on relatively small sample sizes, which increases uncertainty in the reported correlation coefficients and limits the precision of the estimates.

**Figure 2:**
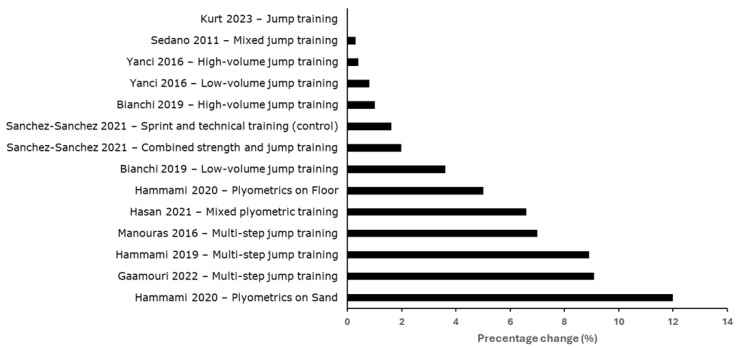
Percentage change in sprint performance following horizontal multi-step jump training interventions [23,24,25,26,27,28,29,30,31,32].

Across the ten intervention studies, several programs reported within-group improvements in short-distance sprint performance, particularly over 5–20 m (Table 3, Figure 2). In some studies, these changes were clearly greater than those observed in control conditions. For example, two studies reported substantial reductions in 5–30 m sprint times in youth handball players, with trivial changes observed in control groups (Table 2) [27,31]. Similarly, larger improvements were reported following sand-based horizontal plyometric training compared with court-based training and control conditions [28].

Other studies reported smaller changes in sprint performance (approximately 1–3% or small absolute time reductions), but these were not different from comparison groups performing alternative plyometric or soccer-based training [23,24,26,29] (Table 3). One study reported no meaningful sprint changes following the intervention [32]. Overall, the most consistent findings were observed for acceleration outcomes (≤20 m), whereas results for longer sprint distances were fewer and more variable across studies. Interventions with the largest improvements typically included multi-step or bounding-based exercises; however, due to variation in program design, training volume, and concurrent training content, no single jump exercise can be identified as consistently more effective across studies (Table 2).

## 4. Discussion

The purpose of this systematic review was to examine the relationship between horizontal multi-step jumps and sprint performance over 0–100 m, and to determine whether training interventions including such exercises improve sprint performance. Across the included studies, horizontal multi-step jump performance showed moderate to strong correlations with sprint performance, although the strength of these relationships varied across populations and sprint distances. However, these findings should be interpreted with caution given the heterogeneity of study designs, variation in sample sizes, and differences in measurement protocols across the included studies. The strongest and most consistent associations were observed for sprint performance within the acceleration phase, whereas weaker or more variable relationships were reported for longer sprint distances [19,22]. Similar patterns were evident in the intervention studies, where improvements were most commonly observed for early-phase sprint performance, particularly over distances ≤20 m [27,28,31].

During the acceleration phase of sprinting, performance is largely determined by the athlete’s ability to generate horizontal impulse over relatively long ground contact times compared with maximal-speed running. Ground contact times during early acceleration are typically reported in the range of ~0.12–0.18 s, allowing greater time for force application in the direction of motion and progressive increases in running velocity across successive steps [1,2]. Horizontal multi-step jump tasks share several of these mechanical characteristics, as they require forward-directed force production and maintenance of horizontal velocity across repeated contacts [5,9]. In contrast, maximal-speed sprinting is characterised by markedly shorter contact times and a greater reliance on vertical force production and leg stiffness [2,4]. This mechanical overlap likely explains why both correlational relationships and training effects involving horizontal multi-step jumps are most consistently observed within the first 20–30 m, whereas associations at longer sprint distances appear weaker or more variable across studies.

Although both multi-steps bounding and single-leg jump tasks are horizontally oriented, differences in execution constraints and contact-time characteristics may help explain why bounding-based tasks tend to show more consistent associations with, and training effects on sprint acceleration than more isolated single-leg tasks. Bounding exercises, particularly when performed with an emphasis on speed, are typically characterized by relatively short and uniform contact times (~0.12–0.18 s) and continuous production of horizontal impulse across successive steps [5,9]. In contrast, single-leg horizontal jump tasks are often associated with longer and more variable contact times, commonly exceeding ~0.18 s and in some cases approaching ~0.25–0.30 s, reflecting greater demands for balance and discrete propulsion rather than continuous velocity maintenance [9,21]. From a mechanical perspective, the continuous nature of bounding more closely resembles sprint acceleration, which may explain why bounding-based tests and interventions more frequently demonstrate moderate-to-strong relationships and meaningful improvements in early sprint performance, whereas findings for isolated single-leg jumps appear more variable across studies and populations.

Differences in athlete background and training status also appear to influence the strength of the observed relationships and training effects between horizontal multi-step jumps and sprint performance. Across the included studies, stronger and more consistent associations were generally reported in trained sprinters and well-trained athletes than in youth or less specialised team-sport populations. For example, studies involving sprinters demonstrated large to very large correlations between horizontal multi-step jump performance and sprint acceleration, whereas weaker or non-significant associations were more commonly observed in younger or less experienced athletes [16,19]. A similar pattern was evident in the intervention studies, where the largest sprint improvements were typically reported in structured training environments with relatively high training loads and clearly defined jump tasks, whereas smaller or non-differential effects were observed in heterogeneous team-sport settings [27,31]. These differences likely reflect variation in technical proficiency, strength levels, and the capacity to exploit horizontally oriented force production during both jumping and sprinting. In younger or less specialised athletes, greater movement variability and ongoing technical development may attenuate both the strength of measured relationships and the magnitude of training-induced sprint adaptations, contributing to the broader range of outcomes observed across studies.

Several methodological factors should be considered when interpreting the findings of this review. The methodological quality assessment indicated that most correlational studies were classified as moderate risk of bias, whereas intervention studies were predominantly classified as low to moderate risk (Table 1), suggesting that the overall findings should be interpreted with appropriate caution. Across the included studies, there was substantial heterogeneity in sprint test distances and outcome definitions, ranging from very short acceleration splits (5–10 m) to longer sprint distances of 40–100 m. Such variation limits direct comparison of effect sizes and correlation magnitudes, particularly given the phase-specific nature of sprint performance. Differences were also evident in the horizontal jump tasks employed, including variations in step number (e.g., 3–10 consecutive contacts), unilateral versus bilateral execution, and whether performance was emphasised toward speed or distance, all of which may influence task mechanics and transfer to sprint outcomes.

In the intervention studies, considerable variability was observed in program design, including intervention duration (typically 6–10 weeks), weekly training frequency (1–3 sessions per week), and total jump volume per session. In several studies, horizontal jump training was combined with other training modalities such as sprint drills, change-of-direction exercises, or strength training, making it difficult to isolate the independent effects of specific horizontal jump exercises. Participant characteristics further contributed to between-study variability, as samples included athletes ranging from early-adolescent to adult age groups and from different sporting backgrounds, factors that are likely to influence both baseline sprint performance and responsiveness to training.

Methodological differences in sprint testing procedures represent an additional source of variability. Although most studies employed electronic timing systems, some relied on contact-based systems or less standardised protocols, which may affect measurement precision, particularly over short sprint distances. Finally, several studies were characterised by relatively small sample sizes (often <25 participants per group), which may reduce statistical power and increase uncertainty in estimated relationships. In addition, several studies originated from overlapping research groups and similar sporting contexts, which may limit the independence of findings and increase the risk of methodological redundancy. As with many narrative syntheses, the possibility of publication bias cannot be excluded, as studies reporting null or trivial findings may be underrepresented in the published literature. Collectively, these methodological considerations highlight the need for caution when comparing results across studies, while not detracting from the consistent overall pattern observed for acceleration-related sprint performance.

This systematic review suggests that horizontal multi-step jumps are generally associated with sprint performance, particularly during the acceleration phase (0–30 m). Across both correlational and intervention studies, repeated horizontal force production appears to be related to early-phase sprint performance, whereas associations and training effects become less consistent as sprint distance increases. While these findings are supported by plausible biomechanical mechanisms, they should be interpreted in light of study heterogeneity and variable methodological quality. Most included studies were classified as moderate risk of bias, and the overall evidence base remains limited by relatively small sample sizes and heterogeneous study designs. Horizontal multi-step jumps may therefore represent useful tools for assessing and potentially developing sprint acceleration but should be integrated alongside other training approaches when targeting maximal running speed. Future research should employ more rigorous experimental designs and larger samples to strengthen the evidence base.

## Figures and Tables

**Table 1:** Risk of bias assessment included correlational and intervention studies.

Study	Design	Score	Risk of Bias
Yanci et al., 2014 [14]	Correlational	6/8	Moderate
Agar-Newman and Klimstra, 2014 [15]	Correlational	6/8	Moderate
Maćkała, et al., 2015 [16]	Correlational	6/8	Moderate
Hasenkamp et al., 2017 [17]	Correlational	6/8	Moderate
Petrakos et al., 2019 [18]	Correlational	6/8	Moderate
Michailidis, 2019 [19]	Correlational	6/8	Moderate
Washif and Kok, 2020 [20]	Correlational	6/8	Moderate
Čoh et al., 2017 [21]	Correlational	6/8	Moderate
Predescu, 2024 [22]	Correlational	6/8	Moderate
Sedano et al., 2011 [23]	Intervention	8/9	Low
Yanci et al., 2015 [24]	Intervention	6/9	Moderate
Manouras et al., 2023 [25]	Intervention	8/9	Low
Bianchi et al., 2019 [26]	Intervention	7/9	Moderate
Hammami et al., 2019 [27]	Intervention	8/9	Low
Hammami et al., 2020 [28]	Intervention	7/9	Moderate
Sanchez-Sanchez et al., 2021 [29]	Intervention	8/9	Low
Hasan et al., 2021 [30]	Intervention	8/9	Low
Gaamouri et al., 2018–2019 [31]	Intervention	7/9	Moderate
Kurt et al., 2023 [32]	Intervention	7/9	Moderate

**Table 2:** Characteristics and reported correlations between horizontal multi-step jump performance and sprint performance.

Study	Participants (n, Sex, Age)	Athlete Type	Jump Tests	Sprint Tests	Main Correlation Outcomes (r)
Yanci et al., 2014 [14]	*n* = 39, male, age 22.9 ± 2.8	Third-division soccer players	Unilateral three-hop jump (H3J) Four-bounce horizontal test (H4BT)	5 m; 10 m; 15 m	H3J and H4BT showed moderate correlations with 5–15 m sprint performance (r = −0.41 to −0.52).
Agar-Newman and Klimstra, 2015 [15]	*n* = 114, female, age 17.9 ± 3.1	Female elite rugby players	Single-leg jump (SLJ); standing triple jump (STJ)	10 m; 40 m sprint	SLJ: r = 0.51 (10 m), r = 0.70 (40 m); STJ: r = 0.61 (10 m), r = 0.75 (40 m).
Maćkała et al., 2015 [16]	*n* = 22, male, age 21.7 ± 1.1	Sprinters and PE students	SLJ; five-jump (SFJ); standing ten-jump (ST10J)	10 m; 30 m	In sprinters, SFJ showed correlations with 10–30 m sprint performance (r = −0.65 to −0.81), and ST10J showed correlations with 10–30 m sprint performance (r = −0.71 to −0.83); negligible correlations were observed in PE students.
Hasenkamp et al., 2017 [17]	*n* = 160, male and female, age 20.0 ± 1.2	Collegiate athletes	Triple hop for distance (THD)	10-yard sprint (9.14 m)	In males, THD showed a correlation with 10-yard sprint performance (r = 0.683); in females, THD showed a correlation with 10-yard sprint performance (r = 0.548).
Petrakos et al., 2019 [18]	*n* = 21, female, age 20.8 ± 1.9	Field sport athletes (hockey, soccer, Gaelic football)	Horizontal jump (HJ); 5-repeated bound (5RB)	0–10 m; 0–20 m	HJ and 5RB showed correlations with sprint performance over 10–20 m (r = −0.47 to −0.56).
Michailidis et al., 2019 [19]	*n* = 19, male, age 14.6 ± 0.8	U15 youth soccer players	Single-leg triple jump (SLTJ)	10 m; 20 m	No significant correlations were observed between horizontal jump asymmetry and sprint performance (SLTJ r = 0.18–0.24).
Washif and Kok, 2020 [20]	*n* = 11, male, age 17.8 ± 1.3	Elite youth sprinters (Malaysia)	Reactive bounding coefficient, 10 bounds (RBC10)	10 m; 30 m; 50 m	RBC10 showed correlations with 10 m sprint performance (r = −0.28) and with 30–50 m sprint performance (r = −0.52 to −0.60).
Čoh et al., 2021 [21]	*n* = 66, male and female, age 21.0 ± 1.6	Multi-sport athletes (football, handball, basketball, tennis, volleyball)	STJ; bilateral triple jump (BTJ25); horizontal single-leg jump over 10 m, left and right (HSLJ10 m L/R); horizontal double-leg jump (HDLJ5); SLJ)	10 m; 30 m	Horizontal multi-hop tests showed correlations with sprint acceleration, with r-values ranging from −0.30 to −0.61 across tests; the highest correlations were observed for HSLJ10 m at 30 m (r = −0.61), followed by STJ (r = −0.60) and BTJ25 (r = −0.56).
Predescu et al., 2025 [22]	*n* = 11, male and female, age 16	Field sprint athletes (U18)	Bounding sprint over 30 m (BS 30 m); STJ	100 m sprint	Horizontal jump performance showed correlations with 100 m sprint time, with r = −0.88 for STJ and r = −0.89 for BS 30 m.

**Table 3:** Characteristics and reported changes in horizontal multi-step jump performance and sprint performance after intervention.

Reference	Sport/Group	Frequence/Duration (Weeks)	Training Group (Description)	Sign. Change	Control Group (Description)	Sign. Change	Between-Group Effect
Sedano et al., 2011 [23]	22 male Young elite U19 soccer players	3/10	A 10-week plyometric program including horizontal multi-hop jumps, hurdle jumps, and lateral jumps	Yes (10 m sprint time decreased by 0.3%)	Followed regular conditioning; trivial change in 10 m sprint	No (0%)	Significant group × time interaction for 10 m sprint (*p* = 0.01)
Yanci et al., 2016 [24]	16 male adult semi-professional soccer players	2/6	Horizontal plyometric training including horizontal repeated countermovement jumps (3-bounce; HRCMJ)	No (0%)	Both groups performed identical plyometric exercises; trivial changes in sprint performance	No (0%)	No significant between-group differences for 5–15 m sprint
Manouras et al., 2016 [25]	30 male adult amateur in-season soccer players	1/8	Horizontal plyometric training including multiple long jumps, long jumps, and diagonal obstacle jumps (60–110 contacts per session)	Yes (30 m sprint time decreased by 2.7%)	Maintained regular soccer training; trivial sprint change	No (0%)	No significant between-group differences at post-test; no effects on 10 m acceleration
Bianchi et al., 2018 [26]	21 Elite youth male football players (Swiss academy)	1–2/8	Single- vs. double-weekly plyometric training; both groups performed horizontal jumps (4 × 6) and single-leg triple hops	Yes (10–40 m sprint time decreased by 0.04–0.21 s in the low-volume plyometric group (LPG) and 0.07–0.09 s in the high-volume plyometric group (HPG)	No control group; two plyometric groups (LPG and HPG) compared	–	No significant group × time interaction for 10–40 m sprint
Hammami et al., 2019 [27]	28 U15 first-division male handball players	2/8	Combined plyometric and short sprint/change of direction (COD) training; Workshops I and III included multidirectional horizontal jumps (3 hops right + 3 left with COD)	Yes (5–30 m sprint time decreased by 7–9%)	Continued standard training; trivial sprint changes	No (0%)	Significant group × time interaction for 5–30 m sprint
Hammami et al., 2020 [28]	31 Junior male handball players	3/7	Plyometric training on sand (PS) or court (P) including 6 horizontal multi-hop jumps (3 left + 3 right) per set	Yes (5–20 m sprint time decreased by 12% in PS and 5% in P)	Followed regular training; minimal sprint improvement	No (0%)	Significant group × time interactions for 5–20 m sprint (PS > P > control)
Sanchez-Sanchez et al., 2021 [29]	20 male National-level amateur soccer players	2/6	A 6-week preseason program; weeks 4–6 included 4 × 5 bilateral horizontal jumps and 2 × 8 unilateral horizontal jumps performed maximally	Yes (15 m sprint time decreased by 2.0%)	Both groups performed the same soccer training; 15 m sprint time decreased by 1.6%	Yes (1.6%)	No significant between-group differences for 15 m sprint (group × time *p* = 0.86)
Hasan et al., 2021 [30]	30 Collegiate male football players	3/6	Plyometric training including bounding (30 m), hurdling, and drop jumps	Yes (50 m sprint time decreased by 6.6% [−0.61 s]	Performed no training; 50 m sprint time decreased by 1.8% (−0.17 s)	Yes (1.8%)	Significant group × time interaction for 50 m sprint
Gaamouri et al., 2023 [31]	28 U17 elite male handball players	2/8	Combined plyometric and high-intensity running training; horizontal jumps included (Workshop III)	Yes (5–30 m sprint time decreased by 7–9%)	Continued regular in-season training; minimal sprint changes	No (0.8%)	Significant group × time interaction for 5–30 m sprint
Kurt et al., 2023 [32]	32 male Adolescent soccer players	2/6	Horizontal plyometric training including bounds, broad jumps, diagonal jumps, and Heidens	No (0%)	Vertical plyometric training (comparison group); no sprint improvement	No (0%)	No significant group × time interactions for 10–20 m sprint

## Data Availability

No new data were created or analyzed in this study.

## Abbreviations

The following abbreviations are used in this manuscript:

BTJ25	Bounding test over 25 m
COD	Change of direction
H3J	Horizontal three-jump test
H4BT	Horizontal four-bounds test
HDLJ5	Horizontal distance five-jump test
HPG	High-volume plyometric group
HRCMJ	Horizontal reactive countermovement jump
HSLJ10m	Horizontal single-leg jump over 10 m
LPG	Low-volume plyometric group
PRISMA	Preferred Reporting Items for Systematic Reviews and Meta-Analyses
RBC10	Reactive bounding coefficient
SLJ	Single-leg jumps
SLTJ	Single-leg triple jump
ST10J	Standing ten-jump test
STJ	Standing triple jump
